# Effects of bone marrow‐derived mesenchymal stromal cells on gene expression in human alveolar type II cells exposed to TNF‐*α*, IL‐1*β*, and IFN‐*γ*

**DOI:** 10.14814/phy2.13831

**Published:** 2018-08-22

**Authors:** Matthew Schwede, Erin M. Wilfong, Rachel L. Zemans, Patty J. Lee, Claudia dos Santos, Xiaohui Fang, Michael A. Matthay

**Affiliations:** ^1^ Department of Medicine University of California San Francisco California; ^2^ Division of Allergy, Pulmonary and Critical Care Medicine Department of Medicine Vanderbilt University Medical Center Nashville Tennessee; ^3^ Division of Pulmonary and Critical Care Medicine University of Michigan Medical School Ann Arbor Michigan; ^4^ Cellular and Molecular Biology Program University of Michigan Medical School Ann Arbor Michigan; ^5^ Section of Pulmonary Critical Care & Sleep Medicine Yale University School of Medicine New Haven Connecticut; ^6^ Interdepartmental Division of Critical Care Medicine St. Michael's Hospital Toronto Ontario Canada; ^7^ Division of Respirology Department of Medicine St. Michael's Hospital Toronto Ontario Canada; ^8^ Li Ka Shing Knowledge Institute Toronto Ontario Canada; ^9^ Cardiovascular Research Institute University of California San Francisco San Francisco California; ^10^ Department of Anesthesia University of California San Francisco San Francisco California

**Keywords:** Acute respiratory distress syndrome, alveolar epithelial cells, cytokines, gene expression profiling, mesenchymal stromal cells

## Abstract

The acute respiratory distress syndrome (ARDS) is common in critically ill patients and has a high mortality rate. Mesenchymal stromal cells (MSCs) have demonstrated therapeutic potential in animal models of ARDS, and their benefits occur in part through interactions with alveolar type II (ATII) cells. However, the effects that MSCs have on human ATII cells have not been well studied. Using previously published microarray data, we performed genome‐wide differential gene expression analyses of human ATII cells that were (1) unstimulated, (2) exposed to proinflammatory cytokines (CytoMix), or (3) exposed to proinflammatory cytokines plus MSCs. Findings were validated by qPCR. Alveolar type II cells differentially expressed hundreds of genes when exposed either to proinflammatory cytokines or to proinflammatory cytokines plus MSCs. Stimulation with proinflammatory cytokines increased expression of inflammatory genes and downregulated genes related to surfactant function and alveolar fluid clearance. Some of these changes, including expression of some cytokines and genes related to surfactant, were reversed by exposure to MSCs. In addition, MSCs induced upregulation of other potentially beneficial genes, such as those related to extracellular matrix remodeling. We confirmed several of these gene expression changes by qPCR. Thus, ATII cells downregulate genes associated with surfactant and alveolar fluid clearance when exposed to inflammatory cytokines, and mesenchymal stromal cells partially reverse many of these gene expression changes.

## Introduction

The acute respiratory distress syndrome (ARDS) is common, affecting approximately 10% of adult intensive care unit patients worldwide (Bellani et al. 2016). With an in‐hospital mortality of almost 40% for ARDS (Rubenfeld et al. 2005) and a lack of disease‐modifying therapies (Laffey and Matthay 2017), developing new treatments are important (Thompson et al. 2017).

Pathologically, ARDS is characterized by injury to the lung parenchyma, which is due in part to an immune response (Laffey and Matthay 2017). Mesenchymal stromal cells (MSCs) have significant therapeutic potential in ARDS through their anti‐inflammatory effects and barrier enhancing effects (Laffey and Matthay 2017; Matthay et al. 2017), and they are currently the focus of clinical trials for ARDS, sepsis, and bronchopulmonary dysplasia. These self‐renewing multipotent stem cells have shown benefits in several preclinical studies (Laffey and Matthay 2017). MSCs reduce pulmonary edema and the inflammatory responses to endotoxin in mouse models of ARDS (Gupta et al. 2007). They improve animal survival in part through the release of proresolving lipids (Fang et al. 2010) and anti‐inflammatory proteins, such as the IL‐1 receptor antagonist (Ortiz et al. 2007). MSCs are known to interact with several types of immune cells (Li et al. 2008; Spaggiari et al. 2006; Zappia et al. 2005). Recently, they were also shown to interact with alveolar type II (ATII) cells (Fang et al. 2010, 2015), attenuating the increased protein permeability and cytoskeletal changes that proinflammatory cytokines induce in cultured ATII cell monolayers (Fang et al. 2010). These effects were mediated by MSC‐dependent angiopoietin‐1 secretion, suggesting a vascular‐mediated mechanism by which they could reduce lung injury and pulmonary edema.

ATII cells make up approximately 2–5% of the alveolar surface area (Jansing et al. 2017) and have several important functions. They produce surfactant, serve as progenitor cells for the alveolar epithelium (Barkauskas et al. 2013) thus helping restore epithelial barriers (Jansing et al. 2017), and play a key role in vectorial sodium‐dependent transport of fluid to maintain dry airspaces (Matthay 2014; Matthay et al. 2002). Progenitor function is regulated by EGFR‐KRAS (Desai et al. 2014), Wnt/*β*‐catenin (Nabhan et al. 2018; Zemans et al. 2011), keratinocyte growth factor (KGF), and hepatocyte growth factor (HGF) (Ware and Matthay 2002) signaling. ATII cells also play a role in host defense through cytokine and chemokine expression (Thorley et al. 2007) and by producing surfactant proteins A and D, which are collectins that opsonize microorganisms (Haczku 2008). However, ATII cell responses both to inflammatory environments and to MSCs are incompletely understood, and their response to MSCs has not been studied on a genome‐wide level.

The objective of this study was to elucidate how MSCs affect epithelial cell biology in an inflammatory environment. Using previously published microarrays (Fang et al. 2015), we performed an unbiased, genome‐wide exploratory analysis of human ATII cell gene expression in response to stimulation with proinflammatory cytokines in the presence or absence of MSCs. We first examined the gene expression of ATII cells exposed to CytoMix (a mixture of TNF‐*α*, IL‐1*β*, and IFN‐*γ*), which models the proinflammatory edema fluid of ARDS (Lee et al. 2007). We also studied the gene expression of ATII cells cocultured with human bone marrow‐derived MSCs and stimulated with CytoMix. We confirmed many of the gene expression changes with qPCR.

In response to CytoMix, ATII cells upregulated cytokines and downregulated genes that code for proteins related to surfactant and alveolar fluid clearance. Exposure to MSCs plus CytoMix partially reversed some of the gene expression changes induced by CytoMix.

## Materials and Methods

### Cell cultures and transwell system

The microarray data used in this study were from a previously described experiment of ATII and MSCs (Fang et al. 2015). The ATII cells for the microarray were isolated from cadaver human lung tissues of five adult males with no lung disease using previously published methods (Fang et al. 2010), and the ATII cells used for quantitative PCR (qPCR) validation were harvested from a different donor using the same methods. The lungs were harvested from brain‐dead subjects, maintained at 4°C, and transported to the University of California, San Francisco within 6 h. Within 24 h of arrival in our laboratory, the ATII cells were isolated from the right middle lobe, if there was no evidence of injury or consolidation to that lobe. Allogeneic human MSCs were obtained from the Tulane Center for Gene Therapy (New Orleans, LA) for microarray studies and from the Institute for Regenerative Medicine at the Texas A&M Health Science Center for qPCR studies. The ATII cells were plated at a density of 1 × 10^6^ cells/well in the upper compartment of Transwell systems (0.4‐mm pore size and collagen I coated; Costar, Corning). The Transwell system was used to study the paracrine effects of MSCs on ATII cells (Huppert and Matthay 2017). As previously described (Fang et al. 2010), the cells were cultured in a 37°C and 5% CO_2_ incubator in mixed media of DMEM high glucose 50% and F‐12 50% containing 10% FBS and antibiotics (gentamicin, penicillin, streptomycin, and amphotericin). Where indicated, CytoMix (IL‐1*β*, TNF‐*α*, and IFN‐*γ*, 50 ng/mL each; R&D Systems) was added to the culture medium, and MSCs were plated in the bottom compartment of the Transwell at a density of 250,000 cells/well with no direct contact with ATII cells, as described previously (Fang et al. 2015). ATII cells, MSCs, and CytoMix were added simultaneously, and conditions were maintained for 24 h (Fang et al. 2015). Three conditions were examined: (1) ATII cells alone (control), (2) ATII cells plus CytoMix, and (3) ATII cells plus MSCs and CytoMix.

### Microarray data processing

The gene expression data used for this study were previously published (Fang et al. 2015) and made publicly available as GSE68610 on the Gene Expression Omnibus (GEO) (Edgar et al. 2002). These data were generated using Affymetrix Human Genome U133A 2.0 Arrays. We downloaded the raw data from GEO and processed them using the Robust Multi‐array Average, or RMA (Irizarry et al. 2003). Only four arrays were available for ATII cells exposed to CytoMix but not MSCs, but five arrays were available for the other conditions. No batch correction was performed because all array experiments were done in a single batch. During data processing, we applied a custom cell description file (CDF) as provided by the Microarray Lab at the University of Michigan (Dai et al. 2005). Custom CDFs were used to ensure that probe set annotations were updated. This method excludes probes that do not match a gene sequence perfectly and uniquely, which may reduce the number of genes used for comparisons (Dai et al. 2005). We used custom CDF version 21 and specifically a CDF that summarizes gene expression data to Entrez Gene Identifiers. We did not exclude any gene based on its level of expression in the dataset. For quality control, we also checked interarray Pearson correlation within each of the three conditions, using a cut‐off of below 0.90 to consider an array for exclusion. Replicate arrays demonstrated within‐condition correlation of at least 0.94.

### Quantitative PCR

Using qPCR, we validated differential expression for genes that had statistically significant expression changes and were of interest because of their biological functions. Total RNA from ATII cells was isolated with the RNeasy Mini kit (Qiagen) and reverse transcribed to cDNA using the “High Capacity RNA‐to‐cDNA Kit” (Applied Biosystems). The assay IDs for TaqMan specific gene primers (Applied Biosystems) were: SFTPB (Hs00167036_m1), SFTPD (Hs01108490_m1), IL23A (Hs00372324_m1), CCL2 (Hs00234140_m1), AQP1 (Hs01028916_m1), AQP3 (Hs00185020_m1), AQP5 (Hs00387048_m1), CXCL10 (Hs00171042_m1), CXCL11 (Hs00171138_m1), POSTN (Hs01566750_m1), LOX (Hs00942480_m1), CASP8 (Hs01018151_m1), SCNN1A (Hs00168906_m1), SCNN1B (Hs01548617_m1), GAPDH (Hs02786624_g1), and EIF2B2 (Hs00204540_m1). qPCR was performed with TaqMan gene primers and TaqMan Fast Advanced Master Mix (Applied Biosystems) using the StepOnePlus System (Applied Biosystems). The average threshold count (Ct) value of three technical replicates was used in all calculations. GAPDH and EIF2B2 were used as a housekeeping genes because they displayed the lowest standard deviation among groups compared to other housekeeping genes tested. Data analysis was performed using the 2^−ΔCt^ method (Schmittgen and Livak 2008). Relative mRNA data are expressed as mean ± standard deviation.

### Statistical analyses

All statistical analyses were performed using R version 3.3.2. We performed principal component analysis using the “prcomp” function in R and hierarchical clustering using tools from the Bioconductor package made4 (Culhane et al. 2005). Gene symbols and descriptions for each gene were generated using the “getBM” function from the biomaRt package in R, and orthologous gene lists between species were generated using the “getLDS” package in biomaRt (Durinck et al. 2009). We excluded arrays that appeared to be outliers based on hierarchical clustering and principal component analysis. We performed differential gene expression analysis using the Bioconductor package limma (Ritchie et al. 2015), and we focused on two comparisons: (1) ATII cell controls versus ATII cells exposed to CytoMix, and (2) ATII cells exposed to CytoMix versus ATII cells exposed to both CytoMix and MSCs. Genes were considered differentially expressed if they were at least twofold differentially expressed between conditions and if the *P*‐value was <0.05 after adjusting for multiple testing using the Benjamini–Hochberg method (Benjamini and Hochberg 1995).

For gene set enrichment analysis of up‐ and downregulated genes, we used gene sets that were downloaded from MSigDB (Subramanian et al. 2005) to look for functional enrichment. *P*‐values for enrichment were generated in R using Fisher's exact test, where the number of genes in our processed microarray dataset was the background number. We adjusted Fisher's exact test *P*‐values for multiple testing using the Benjamini–Hochberg method (Benjamini and Hochberg 1995). We used the “Hallmark” gene sets (Liberzon et al. 2015) from MSigDB in the initial exploratory analyses. “Hallmark” gene sets are derived from published gene sets and describe specific biological states. They were developed in order to reduce heterogeneity and redundancy among descriptive gene sets (Liberzon et al. 2015). We also downloaded the “Canonical pathway” gene sets from MSigDB, which are curated from pathway databases, such as KEGG (Kanehisa and Goto 2000) or Reactome (Croft et al. 2014).

Hierarchical clustering dendrograms and heatmaps were created using the “heatplot” function in the Bioconductor pathway made4 (Culhane et al. 2005). Hierarchical clustering was performed with distance function 1‐Pearson correlation, as previously done (Eisen et al. 1998). We visualized KEGG pathways using the pathview package in Bioconductor (Luo and Brouwer 2013).

## Results

### Mesenchymal stromal cells and CytoMix both modulate ATII cell gene expression

**Figure 1:**
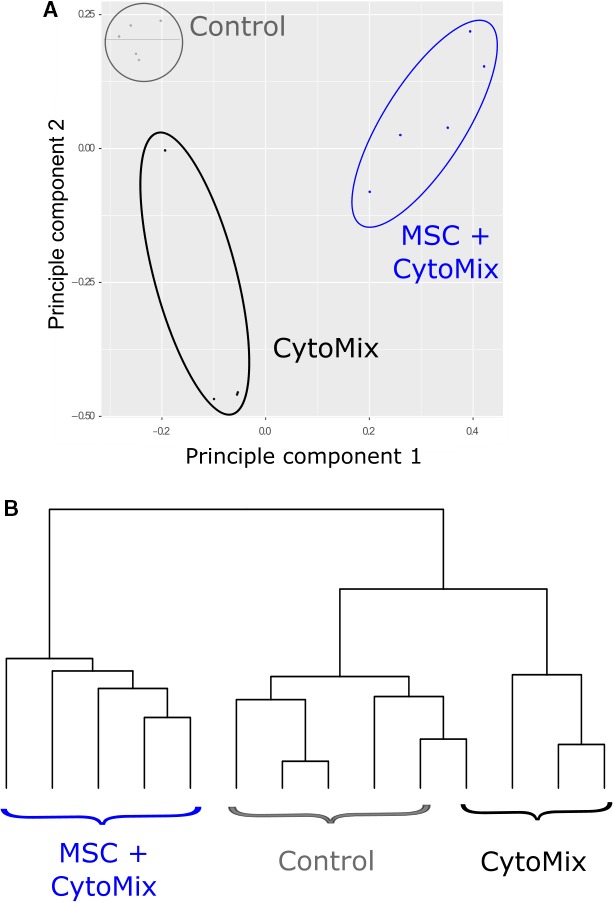
(A) Principle component analysis of all arrays in the type II alveolar cell microarray dataset. (B) Hierarchical clustering of all arrays in the dataset using all genes on the arrays.

We analyzed a total of 12,264 genes in ATII cells derived from human lungs of five individuals. Principle component analysis revealed that most of the gene expression variation (61%) in this dataset could be explained by two principal components (Fig. 1A). The first component (50% of variation) separated ATII cells that were exposed or unexposed to MSCs, and the second (11% of variation) separated ATII cells exposed to only CytoMix from control ATII cells and ATII cells exposed to both CytoMix and MSCs. This suggests that CytoMix and MSCs had independent effects on ATII cell gene expression. For future analyses, we removed one outlier array based on the principal component analysis and hierarchical clustering analysis (Fig. 1). This ATII replicate exposed to only CytoMix clustered with control ATII cells, suggesting that the CytoMix was ineffective in this sample.

### CytoMix induces alveolar type II cells to upregulate proinflammatory genes and downregulate genes required for surfactant production and alveolar fluid clearance

**Figure 2:**
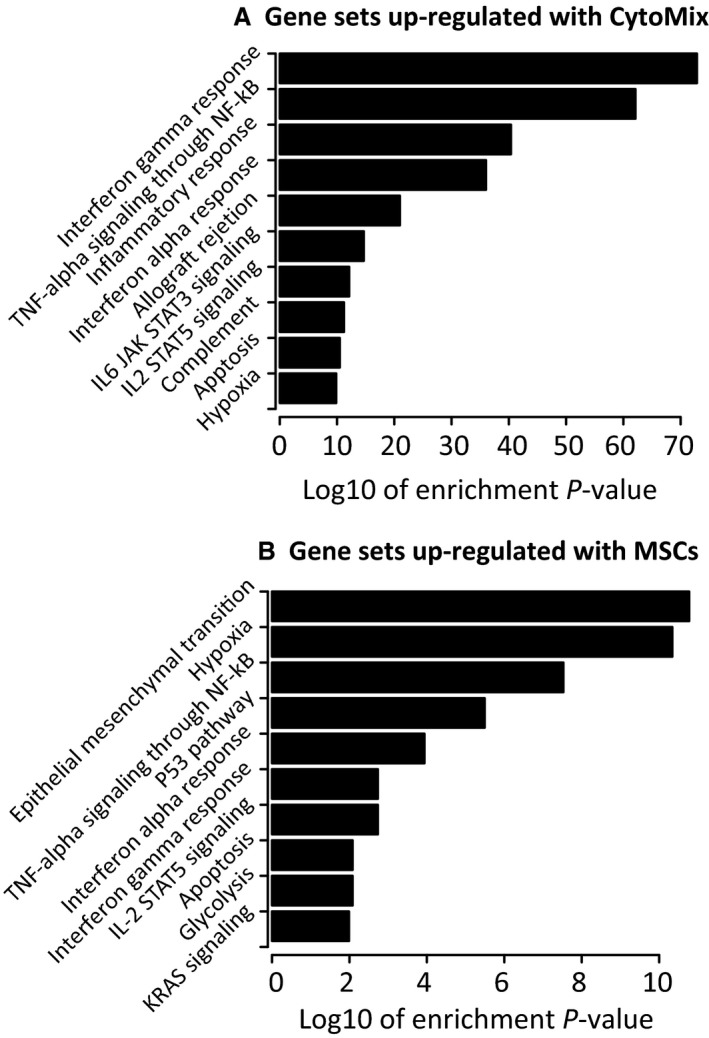
Gene set enrichment of differentially expressed genes. (A) MSigDB Hallmark gene sets upregulated in alveolar type II (ATII) cells exposed to CytoMix. (B) MSigDB Hallmark gene sets upregulated in ATII cells exposed to mesenchymal stromal cells and CytoMix compared to CytoMix alone.

**Figure 3:**
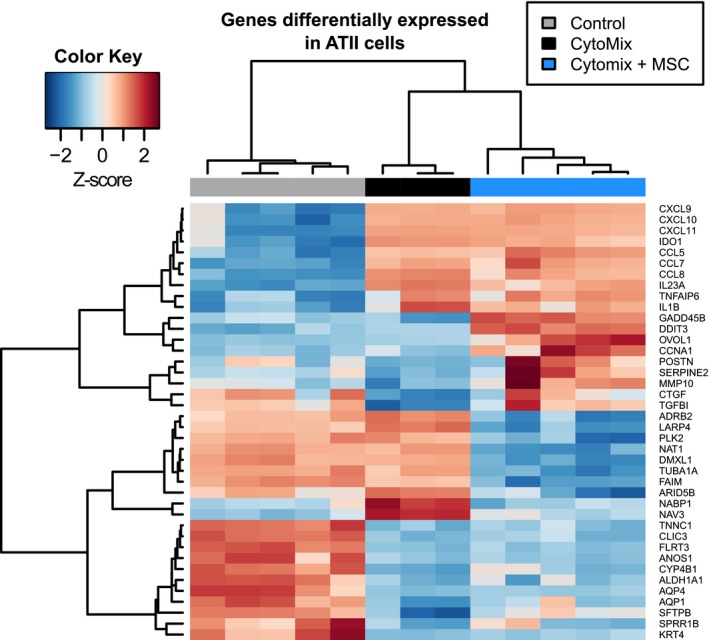
Heatmap of genes differentially expressed by type II alveolar cells (ATII). We included genes differentially expressed when ATII cells were exposed to either CytoMix or to CytoMix plus mesenchymal stromal cells in a Transwell system. The top 10 most up‐ and downregulated genes in each comparison are displayed. Red and blue indicate increased and decreased expression, respectively, normalized to a *z*‐score for each gene.

**Figure 4:**
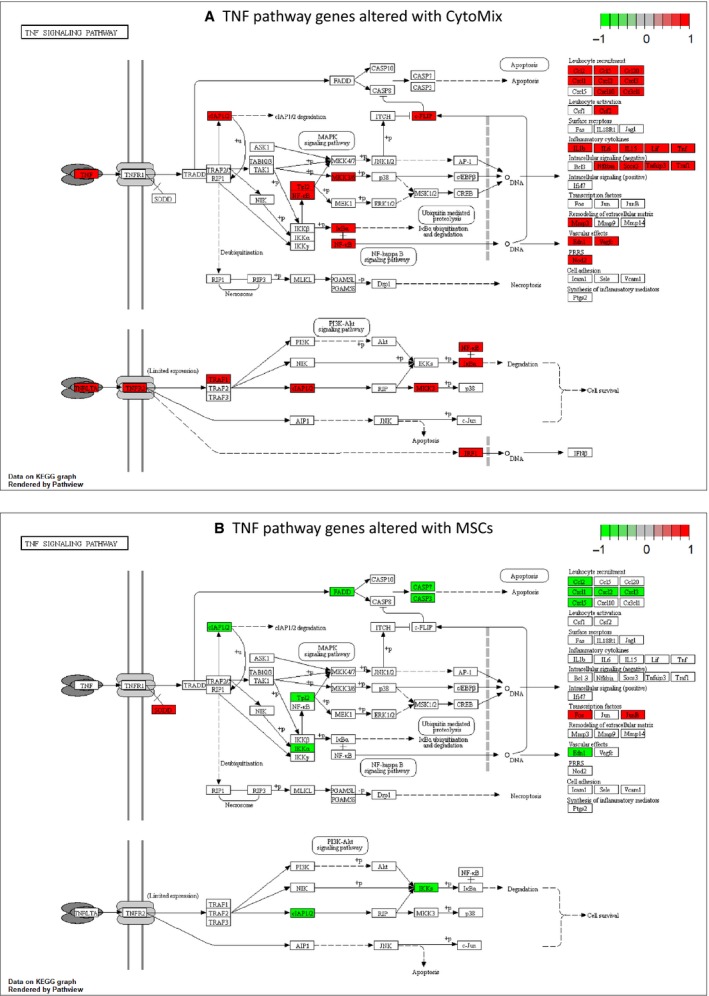
Differential expression of TNF pathway genes. Figures were generated using the “pathview” package in Bioconductor (Luo and Brouwer 2013). Red means upregulated and green means downregulated. (A) Genes from the KEGG (Kanehisa and Goto 2000) TNF pathway that are differentially expressed in ATII cells exposed to CytoMix compared to control. (B) Genes from the KEGG TNF pathway that are differentially expressed in ATII cells exposed to mesenchymal stromal cells and CytoMix compared to CytoMix alone.

A total of 409 and 454 genes were up‐ and down regulated, respectively, in ATII cells in response to CytoMix (Table S1). Upregulated genes were enriched for several MSigDB Hallmark gene sets and pathways related to inflammation (Fig. 2, Table S2). These include gene sets describing response to TNF‐*α* via NF‐*κ*B signaling (enrichment adjusted *P*‐value 8 × 10^−63^) and response to interferon gamma (*P* = 1 × 10^−73^). For example, the most strongly upregulated genes included those encoding chemokines CXCL10 and CXCL11 (Fig. 3), both of which are upregulated over 100‐fold and are known to be induced by interferon gamma (Kanda et al. 2007). Genes coding CCL5, CCL7, and CCL8 were also upregulated, as was IL1*β*. Of the canonical pathways, the Reactome cytokines pathway is the most statistically significantly upregulated (*P *= 2 × 10^−20^, Table S1). Using the “pathview” function in Bioconductor (Luo and Brouwer 2013), we also visualized up‐ and downregulated genes within the KEGG TNF pathway. Many elements of the TNF pathway were upregulated, as were numerous cytokines and chemokines (Fig. 4).

Gene sets downregulated in ATII cells following exposure to CytoMix were related to lipid metabolism and alveolar fluid transport. The most enriched MSigDB Hallmark gene set was adipogenesis (***P*** = 4 × 10^−6^, Table S3), which includes genes coding for proteins important for pulmonary surfactant function, such as long chain Acyl‐CoA dehydrogenase (Goetzman et al. 2014). Surfactant proteins B and D were downregulated 23‐ and 10‐fold, respectively (Fig. 3). Downregulated genes also included those involved in ion and water channel function (Fig. 3, Table S1). The alpha and beta subunits of epithelial sodium channel (ENaC) were not differentially expressed in the initial genome‐wide analysis adjusted for multiple testing. However, given the importance of the ENaC channel to alveolar fluid clearance (Matthay 2014), we carried out focused, hypothesis‐driven statistical testing without multiple testing adjustment. We found that genes encoding both ENaC alpha and beta subunits were downregulated (***P*** = 6 × 10^−5^ and 0.03, respectively).

### Mesenchymal stromal cells alter multiple aspects of ATII gene expression

Exposure of ATII cells to CytoMix plus MSCs resulted in the upregulation of 215 and downregulation of 938 genes compared to CytoMix alone (Table S4).

Functional enrichment analysis identified various upregulated genes involved in epithelial‐mesenchymal transition (***P*** = 2 × 10^−11^, Fig. 2, Table S5). For example, we identified upregulation of SNAIL (adjusted ***P*** = 0.005), and downregulation of E‐cadherin (adjusted ***P*** = 0.001). However, several EMT genes (Lamouille et al. 2014) were not differentially expressed, such as genes coding Twist, Vimentin, MMP2, MMP9, N‐cadherin, ZEB1, or ZEB2.

Among the genes in this Hallmark gene set, two of the most strongly upregulated were genes coding for periostin and lysyl oxidase (Table S4), proteins involved in collagen cross‐linking (Maruhashi et al. 2010) (Fig. 3), which suggests extracellular matrix modification. Consistently, the canonical pathways that were most enriched in these upregulated genes included several pathways related to the extracellular matrix (Table S5). Genes coding for fibronectin and matrix metalloproteinases were also strongly upregulated, consistent with wound healing and repair.

To further analyze the ATII cells' reparative potential, we examined differential expression of other factors, such as EGFR (Desai et al. 2014) and genes related to Wnt signaling (Nabhan et al. 2018; Zemans et al. 2011). Although several of these genes did not meet our original cutoff of fold‐change >2, EGFR, WNT2, WNT8B, WNT10B, WNT11, and WNT16 were all significantly upregulated (***P*** < 0.05, after multiple testing adjustment) with MSC exposure. Some genes related to proliferation, such as KI67, E2F1, E2F2, and E2F4, were similarly upregulated, although other genes related to proliferation, such as MYC signaling (Table S7), were downregulated. Lastly, we noted increased expression of angiopoeitin‐1, which helps restore more normal ATII cell paracellular permeability to protein (Fang et al. 2010), and nearly significantly increased HGF expression (***P*** = 0.052), which also helps with repair (Ware and Matthay 2002).

Although MSC exposure was associated with upregulation of some genes related to TNF‐*α* signaling (Fig. 2, Table S5), the specific pathway genes were different from those upregulated with CytoMix exposure (Table S6). Figure 4 shows that several genes from the KEGG TNF pathway were downregulated with MSC exposure, particularly chemokines, suggesting that MSCs have an anti‐inflammatory effect. Genes associated with apoptosis such those coding for FADD and caspase proteins and the Reactome apoptosis pathway (Table S7) were also downregulated, suggesting an antiapoptotic effect of MSCs on ATII cells. Similarly, genes coding for other antiapoptotic proteins, such as SODD or CHOP, were upregulated, with *DDIT3*, the gene that codes for CHOP, being eightfold increased and the fifth most strongly upregulated gene. The TNF‐*α* pathway genes upregulated by MSCs but not by CytoMix also included several coding for transcription factors such as HES1, FOS, JUNB, and KLF2, the last of which is essential for type 1 pneumocyte differentiation during development in mice (Pei et al. 2011), a process also important for lung injury repair (Jansing et al. 2017).

### MSCs attenuate inflammatory changes induced by CytoMix

In genome wide analyses adjusted for multiple testing, genes upregulated by CytoMix were significantly downregulated by MSCs (Fisher's test *P* = 7 × 10^−11^), and those downregulated by CytoMix were also significantly upregulated by MSCs (*P* = 6 × 10^−9^). Genes with transcriptional changes induced by CytoMix and reversed by exposure to MSCs included those coding surfactant protein B, IL‐23, and CCL2, which is a chemokine involved in ARDS pathogenesis (Williams et al. 2017) (Table S8). In hypothesis‐driven tests for genes that we found were downregulated by CytoMix, we also found that the alpha and beta subunits of ENaC were upregulated with MSC exposure (*P* = 0.01 and 4 × 10^−5^, respectively).

Notably, not all genes related to inflammation were reversed following 24 h of MSC exposure. For example, ATII cells are known to express pattern recognition receptors (PRR) (Evans et al. 2010); several of these were upregulated by CytoMix but their expression did not change in response to MSCs. For example, NOD2, which is a PRR expressed by epithelial cells (Uehara et al. 2007), was upregulated over 3‐fold in response to CytoMix and remained upregulated in cells exposed to CytoMix plus MSCs. A similar pattern was observed for *DDX58*, the gene coding for the PRR RIG‐I. The only Toll‐like receptor (TLR) that was downregulated with MSC exposure in our initial analysis was TLR3 (Table S4).

### Comparison of MSC effects to those from a mouse model

In order to validate these differentially expressed genes, we examined comparable differentially expressed genes from a mouse model (dos Santos et al. 2012). In dos Santos et al. (2012), mice underwent cecal ligation with or without exposure to MSCs, and differential gene expression analysis of several murine tissues, including the lung, was performed. We found that 33% of the genes that were differentially expressed in ATII cells exposed to MSCs (Table S4) overlapped with those from dos Santos et al. (Fisher's test *P* = 0.0004), suggesting that the MSCs affected similar pathways in both models. The downregulated genes had particularly strong overlap with those downregulated in dos Santos et al. (19%, *P* = 4 × 10^−6^), with similar downregulation of caspase 3 and chemokine ligands CXCL1, CXCL2, CXCL3, and CCL4. The upregulated genes were under‐enriched in those upregulated in dos Santos et al. (4%, *P* = 0.005).

### Validation with quantitative PCR

**Figure 5:**
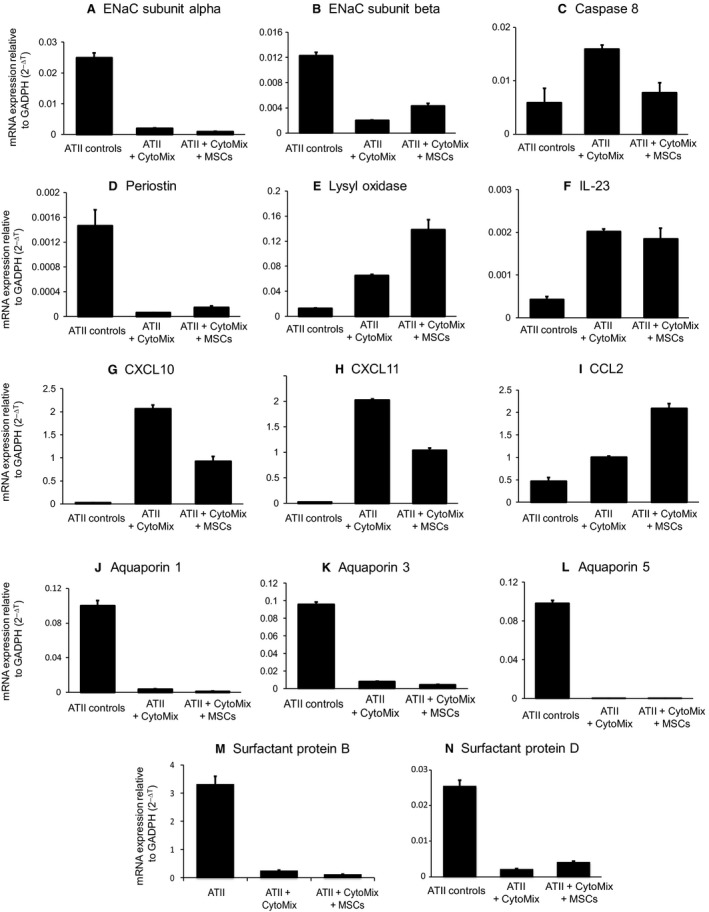
Quantitative PCR validation. Gene expression changes in ATII cells for several genes across three conditions: control, exposure to CytoMix, and exposure to both CytoMix and MSCs. Gene expression values are from qPCR, normalized to reference gene GAPDH. The genes featured are those coding for (A) ENaC subunit alpha, (B) ENaC subunit beta, (C) Caspase 8, (D) Periostin, (E) Lysyl oxidase, (F) IL‐23, (G) CXCL10, (H) CXCL11, (I) CCL2, (J) Aquaporin 1, (K) Aquaporin 3, (L) Aquaporin 5, (M) Surfactant protein B, (N) Surfactant protein D.

For the genes tested, all gene expression changes induced by CytoMix were confirmed with qPCR (Fig. 5). CytoMix induced upregulation of genes coding for several cytokines, including CXCL10, CXCL11, CCL2, and IL‐23, and it induced downregulation of genes coding for surfactant protein B, surfactant protein D, and the ENaC subunits alpha and beta.

The effects of MSCs were not all confirmed with qPCR, but the gene expression trends were overall similar to those of the microarray. Exposure to MSCs resulted in downregulation of CXCL10, CXCL11, and caspase 8, but IL‐23 was not substantially downregulated, and CCL2 was actually upregulated with MSC exposure by qPCR. Additionally, surfactant protein D and ENaC beta expression increased with MSC exposure, but surfactant protein B and ENaC alpha expression remained unchanged. MSCs caused increased gene expression of lysyl oxidase and periostin, similar to the microarray results. Interestingly, in the microarray analysis, surfactant protein D, CXCL10, and CXCL11 were not differentially expressed with MSC plus CytoMix compared to CytoMix alone (*P* > 0.10 for those comparisons).

## Discussion

The primary findings of this study can be summarized as follows. Several gene expression pathways in ATII cells were modified in response to a mixture of TNF‐*α*, IL‐1*β*, and IFN‐*γ*, which models the proinflammatory edema fluid of ARDS (Lee et al. 2007). MSCs reversed some, but not all of the effects of CytoMix on ATII cells, including upregulation of inflammatory genes and downregulation of genes related to key ATII cell functions, such as surfactant production and alveolar fluid clearance.

ATII cells exposed to CytoMix upregulated genes coding several cytokines and proapoptotic proteins. It has been previously shown that ATII cells can express cytokines in infection or inflammatory environments (Stegemann‐Koniszewski et al. 2016). In response to CytoMix, ATII cells also downregulated genes important for normal ATII cell function, such as genes related to surfactant and vectorial alveolar fluid transport. Consistently, previous studies have shown that the inflammatory environment induced by endotoxin reduced the surfactant production in mice (Islam et al. 2012) and alveolar fluid clearance in the human lung (Lee et al. 2009). In addition, several genes related to lipid production and genes encoding surfactant B, which is important for maintaining alveolar tension (Whitsett et al. 1995), and surfactant D, which is thought to support host defense in the alveolus (Hartl and Griese 2006), were downregulated. Genes important for fluid transport were also downregulated, such as those coding for the alpha and beta subunits of ENaC. CytoMix was previously shown to induce downregulation of ENaC subunits alpha and beta in ATII cells (Lee et al. 2007).

In contrast, MSCs induced ATII cells to reverse some of the gene expression changes induced by CytoMix. With MSCs, ATII cells upregulated the genes coding surfactant protein D and ENaC subunit beta while they downregulated gene expression for genes associated with apoptosis, such as caspase 8, and cytokines, such as CXCL10, which has been linked to ARDS pathogenesis (Ichikawa et al. 2013).

Our research group has previously reported that MSCs partially restore fluid clearance in injured lung through ENaC‐mediated sodium transport (Lee et al. 2009). While the effect of MSCs on ENaC alpha did not validate with qPCR, ENaC activity in alveolar cells may also change independent of gene or protein expression (Planès et al. 2002). The same is true for caspases, where posttranslational modification alters their catalytic activity (Zamaraev et al. 2017).

MSCs also induced ATII cells to upregulate several genes, including those in pathways related to injury repair and the epithelial–mesenchymal transition. The significance of epithelial–mesenchymal transition genes is unclear because many other genes associated with epithelial–mesenchymal transition were not differentially expressed. However, the most strongly upregulated of these genes were related to extracellular matrix modification. For example, ATII cells exposed to MSCs upregulated the gene coding for periostin, which has been shown to enhance wound repair in alveolar epithelial cells (Akram et al. 2013). Periostin also causes airway fibroblasts to overexpress collagen and helps stiffen the collagen matrix (Sidhu et al. 2010), making it potentially beneficial for epithelial repair. Consistently, several genes related to repair, such as EGFR, angiopoietin‐1, and HGF were also statistically significantly up‐regulated.

MSCs did not reverse all genes associated with CytoMix exposure, including genes that could be relevant for host defense, such as PRR. TLR3 was downregulated with MSC exposure, but this may be advantageous since TLR3‐deficient mice with influenza have a survival advantage, potentially because of an attenuated inflammatory response (Le Goffic et al. 2006).

Most of our findings validated with qPCR, which is expected using arrays and data processing methods similar to our own (Dallas et al. 2005). In our experiment, a few gene expression changes in the microarray analyses were not validated by qPCR, particularly those related to MSC exposure. For example, with MSC exposure, chemokine CCL2 gene expression increased in the qPCR analysis but decreased in the microarray analysis. Several factors may have contributed to discrepancies between microarray and qPCR, including biological variability. The qPCR was performed using a new ATII biological replicate, and genes used for qPCR were in part chosen because of biological interest, not always because of magnitude of differential expression. The qPCR studies were also performed using MSCs derived from an individual different from those in the original microarray. Regardless, most of the trends related to MSC exposure were confirmed with qPCR, such as reduced ATII cytokine gene expression and increased gene expression associated with alveolar fluid clearance and surfactant. It is also possible that different MSC experiments alter similar transcriptional programs but potentially different genes. This is further supported by the significant overlap of these genes differentially expressed in ATII cells and genes differentially expressed in a mouse model of sepsis, in which mice were also exposed to MSCs (dos Santos et al. 2012).

Our study has several advantages. Genome‐wide analysis of human ATII cells exposed to human mesenchymal stromal cells in an inflammatory milieu is novel and allowed us to identify previously unexplored changes in ATII cells. Our study offers the added benefit of enriching for the low abundance ATII cells, compared to whole‐tissue expression analyses (Stone et al. 1992). We also studied human rather than animal cells. The volume of data offered by our genome‐wide approach revealed several ATII cell adaptations to both inflammation and MSCs, which sets the stage for mechanistic experiments.

The limitations to these studies include the in vitro approach, the modest sample size, the lack of an MSC‐only group, and the reliance on gene expression analyses. The in vitro Transwell model has been useful in studies of alveolar cell protein permeability (Fang et al. 2010), alveolar fluid clearance (Lee et al. 2007), and interactions with MSCs (Fang et al. 2010, 2015). In addition, the proinflammatory cytokines in this study reproduce many of the effects of pulmonary edema fluid from ARDS patients (Lee et al. 2007), and in vivo experiments have generated complementary results to Transwell experiments (Fang et al. 2015). However, the environment encountered by ATII cells in vivo is more complex, with inflammatory cells, endothelial cells, and lymphatics, which were not accounted for in this study. In the future, the genes identified here as upregulated by ATII cells in response to MSCs could be inhibited in in vivo models of lung injury to confirm that these genes indeed mediate the protective effect of MSCs in vivo. Although the sample size is limited, we were able to discover statistically significantly differentially expressed genes that had at least twofold change across biological replicates of ATII cells. Because we did not have a group of MSC‐only condition, we could not distinguish the effects of MSCs on ATII cells from the interaction of MSCs and CytoMix on ATII cells. However, the comparison of MSCs plus an inflammatory environment versus the inflammatory environment alone is the most clinical relevant comparison and has been previously used (dos Santos et al. 2012). Lastly, our study only explored mRNA expression changes, and future studies could include protein or micro RNA studies to further validate and explore these changes induced by MSCs.

This study shows that exposure to an acute inflammatory environment induces ATII cells to upregulate genes that code cytokines and downregulate those associated with alveolar fluid clearance and surfactant. Exposure to MSCs partially reversed several of these changes.

## Conflict of Interest

No conflicts of interest, financial or otherwise, are declared by the authors.

## Data Accessibility

## Supporting information




**Table S1.** Genes differentially expressed by ATII cells that were exposed to CytoMix alone versus control.
**Table S2.** MSigDB Hallmark gene sets and canonical pathway gene sets upregulated in ATII cells that were exposed to CytoMix alone versus. control.
**Table S3.** MSigDB Hallmark gene sets and canonical pathway gene sets downregulated in ATII cells that were exposed to CytoMix alone versus control.
**Table S4.** Genes differentially expressed by ATII cells that were exposed to MSCs plus CytoMix versus CytoMix alone.
**Table S5.** MSigDB Hallmark gene sets and canonical pathway gene sets upregulated in ATII cells that were exposed to MSCs plus CytoMix versus CytoMix alone.
**Table S6.** TNF‐α signaling genes that are upregulated with CytoMix or MSC exposure.
**Table S7.** MSigDB Hallmark gene sets and canonical pathway gene sets downregulated in ATII cells that were exposed to MSCs and CytoMix compared to CytoMix alone.
**Table S8.** Genes whose expression pattern reverses with MSC exposure in the microarray.

## References

[phy213831-bib-0001] Akram, K. M. , S. Samad , M. A. Spiteri , and N. R. Forsyth . 2013 Mesenchymal stem cells promote alveolar epithelial cell wound repair in vitro through distinct migratory and paracrine mechanisms. Respir. Res. 14:9. 23350749 10.1186/1465-9921-14-9 PMC3598763

[phy213831-bib-0002] Barkauskas, C. E. , M. J. Cronce , C. R. Rackley , E. J. Bowie , D. R. Keene , B. R. Stripp , et al. 2013 Type 2 alveolar cells are stem cells in adult lung. J. Clin. Invest. 123:3025–3036. 23921127 10.1172/JCI68782 PMC3696553

[phy213831-bib-0003] Bellani, G. , J. G. Laffey , T. Pham , E. Fan , L. Brochard , A. Esteban , et al. 2016 Epidemiology, patterns of care, and mortality for patients with acute respiratory distress syndrome in intensive care units in 50 countries. JAMA 315:788–800. 26903337 10.1001/jama.2016.0291

[phy213831-bib-0004] Benjamini, Y. , and Y. Hochberg . 1995 Controlling the false discovery rate: A practical and powerful approach to multiple testing. J. R Stat. Soc. Ser. B Methodol. 57:289–300.

[phy213831-bib-0005] Croft, D. , A. F. Mundo , R. Haw , M. Milacic , J. Weiser , G. Wu , et al. 2014 The reactome pathway knowledgebase. Nucleic Acids Res. 42:D472–D477. 24243840 10.1093/nar/gkt1102 PMC3965010

[phy213831-bib-0006] Culhane, A. C. , J. Thioulouse , G. Perrière , and D. G. Higgins . 2005 MADE4: an R package for multivariate analysis of gene expression data. Bioinformatics 21:2789–2790. 15797915 10.1093/bioinformatics/bti394

[phy213831-bib-0007] Dai, M. , P. Wang , A. D. Boyd , G. Kostov , B. Athey , E. G. Jones , et al. 2005 Evolving gene/transcript definitions significantly alter the interpretation of GeneChip data. Nucleic Acids Res. 33:e175. 16284200 10.1093/nar/gni179 PMC1283542

[phy213831-bib-0008] Dallas, P. B. , N. G. Gottardo , M. J. Firth , A. H. Beesley , K. Hoffmann , P. A. Terry , et al. 2005 Gene expression levels assessed by oligonucleotide microarray analysis and quantitative real‐time RT‐PCR – how well do they correlate? BMC Genom. 6:59. 10.1186/1471-2164-6-59 PMC1142514 15854232

[phy213831-bib-0009] Desai, T. J. , D. G. Brownfield , and M. A. Krasnow . 2014 Alveolar progenitor and stem cells in lung development, renewal and cancer. Nature 507:190–194. 24499815 10.1038/nature12930 PMC4013278

[phy213831-bib-0010] Durinck, S. , P. T. Spellman , E. Birney , and W. Huber . 2009 Mapping identifiers for the integration of genomic datasets with the R/Bioconductor package biomaRt. Nat. Protoc. 4:1184–1191. 19617889 10.1038/nprot.2009.97 PMC3159387

[phy213831-bib-0011] Edgar, R. , M. Domrachev , and A. E. Lash . 2002 Gene expression omnibus: NCBI gene expression and hybridization array data repository. Nucleic Acids Res. 30:207–210. 11752295 10.1093/nar/30.1.207 PMC99122

[phy213831-bib-0012] Eisen, M. B. , P. T. Spellman , P. O. Brown , and D. Botstein . 1998 Cluster analysis and display of genome‐wide expression patterns. Proc. Natl Acad. Sci. USA 95:14863–14868. 9843981 10.1073/pnas.95.25.14863 PMC24541

[phy213831-bib-0013] Evans, S. E. , Y. Xu , M. J. Tuvim , and B. F. Dickey . 2010 Inducible innate resistance of lung epithelium to infection. Annu. Rev. Physiol. 72:413–435. 20148683 10.1146/annurev-physiol-021909-135909 PMC4471865

[phy213831-bib-0014] Fang, X. , A. P. Neyrinck , M. A. Matthay , and J. W. Lee . 2010 Allogeneic human mesenchymal stem cells restore epithelial protein permeability in cultured human alveolar type II cells by secretion of angiopoietin‐1. J. Biol. Chem. 285:26211–26222. 20554518 10.1074/jbc.M110.119917 PMC2924032

[phy213831-bib-0015] Fang, X. , J. Abbott , L. Cheng , J. K. Colby , J. W. Lee , B. D. Levy , et al. 2015 Human mesenchymal stem (Stromal) cells promote the resolution of acute lung injury in part through lipoxin A4. J. Immunol. 1950:875–881. 10.4049/jimmunol.1500244 26116507

[phy213831-bib-0016] Goetzman, E. S. , J. F. Alcorn , S. S. Bharathi , R. Uppala , K. J. McHugh , B. Kosmider , et al. 2014 Long‐chain acyl‐CoA dehydrogenase deficiency as a cause of pulmonary surfactant dysfunction. J. Biol. Chem. 289:10668–10679. 24591516 10.1074/jbc.M113.540260 PMC4036448

[phy213831-bib-0017] Gupta, N. , X. Su , B. Popov , J. W. Lee , V. Serikov , and M. A. Matthay . 2007 Intrapulmonary delivery of bone marrow‐derived mesenchymal stem cells improves survival and attenuates endotoxin‐induced acute lung injury in mice. J. Immunol. 1950:1855–1863. 10.4049/jimmunol.179.3.1855 17641052

[phy213831-bib-0018] Haczku, A . 2008 Protective role of the lung collectins surfactant protein A and surfactant protein D in airway inflammation. J. Allergy Clin. Immunol. 122: 861–879; quiz 880–881. 19000577 10.1016/j.jaci.2008.10.014 PMC4097097

[phy213831-bib-0019] Hartl, D. , and M. Griese . 2006 Surfactant protein D in human lung diseases. Eur. J. Clin. Invest. 36:423–435. 16684127 10.1111/j.1365-2362.2006.01648.x

[phy213831-bib-0020] Huppert, L. A. , and M. A. Matthay . 2017 Alveolar fluid clearance in pathologically relevant conditions: in vitro and in vivo models of acute respiratory distress syndrome. Front Immunol. 8: 371. 28439268 10.3389/fimmu.2017.00371 PMC5383664

[phy213831-bib-0021] Ichikawa, A. , K. Kuba , M. Morita , S. Chida , H. Tezuka , H. Hara , et al. 2013 CXCL10‐CXCR3 enhances the development of neutrophil‐mediated fulminant lung injury of viral and nonviral origin. Am. J. Respir. Crit. Care Med. 187:65–77. 23144331 10.1164/rccm.201203-0508OC PMC3927876

[phy213831-bib-0022] Irizarry, R. A. , B. Hobbs , F. Collin , Y. D. Beazer‐Barclay , K. J. Antonellis , U. Scherf , et al. 2003 Exploration, normalization, and summaries of high density oligonucleotide array probe level data. Biostatistics 4:249–264. 12925520 10.1093/biostatistics/4.2.249

[phy213831-bib-0023] Islam, M. N. , S. R. Das , M. T. Emin , M. Wei , L. Sun , K. Westphalen , et al. 2012 Mitochondrial transfer from bone‐marrow‐derived stromal cells to pulmonary alveoli protects against acute lung injury. Nat. Med. 18:759–765. 22504485 10.1038/nm.2736 PMC3727429

[phy213831-bib-0024] Jansing, N. L. , J. McClendon , P. M. Henson , R. M. Tuder , D. M. Hyde , and R. L. Zemans . 2017 Unbiased quantitation of alveolar type II to alveolar type I cell transdifferentiation during repair after lung injury in mice. Am. J. Respir. Cell Mol. Biol. 57:519–526. 28586241 10.1165/rcmb.2017-0037MA PMC5705906

[phy213831-bib-0025] Kanda, N. , T. Shimizu , Y. Tada , and S. Watanabe . 2007 IL‐18 enhances IFN‐gamma‐induced production of CXCL9, CXCL10, and CXCL11 in human keratinocytes. Eur. J. Immunol. 37:338–350. 17274000 10.1002/eji.200636420

[phy213831-bib-0026] Kanehisa, M. , and S. Goto . 2000 KEGG: kyoto encyclopedia of genes and genomes. Nucleic Acids Res. 28:27–30. 10592173 10.1093/nar/28.1.27 PMC102409

[phy213831-bib-0027] Laffey, J. G. , and M. A. Matthay . 2017 Fifty years of research in ARDS. Cell‐based therapy for acute respiratory distress syndrome. Biology and potential therapeutic value. Am. J. Respir. Crit. Care Med. 196:266–273. 28306336 10.1164/rccm.201701-0107CP PMC5549868

[phy213831-bib-0028] Lamouille, S. , J. Xu , and R. Derynck . 2014 Molecular mechanisms of epithelial‐mesenchymal transition. Nat. Rev. Mol. Cell Biol. 15:178–196. 24556840 10.1038/nrm3758 PMC4240281

[phy213831-bib-0029] Le Goffic, R. , V. Balloy , M. Lagranderie , L. Alexopoulou , N. Escriou , R. Flavell , et al. 2006 Detrimental contribution of the Toll‐like receptor (TLR)3 to influenza A virus‐induced acute pneumonia. PLoS Pathog. 2:e53. 16789835 10.1371/journal.ppat.0020053 PMC1475659

[phy213831-bib-0030] Lee, J. W. , X. Fang , G. Dolganov , R. D. Fremont , J. A. Bastarache , L. B. Ware , et al. 2007 Acute lung injury edema fluid decreases net fluid transport across human alveolar epithelial type II cells. J. Biol. Chem. 282:24109–24119. 17580309 10.1074/jbc.M700821200 PMC2765119

[phy213831-bib-0031] Lee, J. W. , X. Fang , N. Gupta , V. Serikov , and M. A. Matthay . 2009 Allogeneic human mesenchymal stem cells for treatment of *E. coli* endotoxin‐induced acute lung injury in the ex vivo perfused human lung. Proc. Natl Acad. Sci. USA 106:16357–16362. 19721001 10.1073/pnas.0907996106 PMC2735560

[phy213831-bib-0032] Li, Y.‐P. , S. Paczesny , E. Lauret , S. Poirault , P. Bordigoni , F. Mekhloufi , et al. 2008 Human mesenchymal stem cells license adult CD34^+^ hemopoietic progenitor cells to differentiate into regulatory dendritic cells through activation of the Notch pathway. J. Immunol. 1950:1598–1608. 10.4049/jimmunol.180.3.1598 18209056

[phy213831-bib-0033] Liberzon, A. , C. Birger , H. Thorvaldsdóttir , M. Ghandi , J. P. Mesirov , and P. Tamayo . 2015 The molecular signatures database hallmark gene set collection. Cell Syst. 1:417–425. 26771021 10.1016/j.cels.2015.12.004 PMC4707969

[phy213831-bib-0034] Luo, W. , and C. Brouwer . 2013 Pathview: an R/Bioconductor package for pathway‐based data integration and visualization. Bioinformatis 29:1830–1831. 10.1093/bioinformatics/btt285 PMC3702256 23740750

[phy213831-bib-0035] Maruhashi, T. , I. Kii , M. Saito , and A. Kudo . 2010 Interaction between periostin and BMP‐1 promotes proteolytic activation of lysyl oxidase. J. Biol. Chem. 285:13294–13303. 20181949 10.1074/jbc.M109.088864 PMC2857065

[phy213831-bib-0036] Matthay, M. A. 2014 Resolution of pulmonary edema. Thirty years of progress. Am. J. Respir. Crit. Care Med. 189:1301–1308. 24881936 10.1164/rccm.201403-0535OE PMC4098087

[phy213831-bib-0037] Matthay, M. A. , H. G. Folkesson , and C. Clerici . 2002 Lung epithelial fluid transport and the resolution of pulmonary edema. Physiol. Rev. 82:569–600. 12087129 10.1152/physrev.00003.2002

[phy213831-bib-0038] Matthay, M. A. , S. Pati , and J.‐W. Lee . 2017 Concise review: mesenchymal stem (Stromal) cells: biology and preclinical evidence for therapeutic potential for organ dysfunction following trauma or sepsis. Stem Cells 35:316–324. 27888550 10.1002/stem.2551

[phy213831-bib-0039] Nabhan, A. N. , D. G. Brownfield , P. B. Harbury , M. A. Krasnow , and T. J. Desai . 2018 Single‐cell Wnt signaling niches maintain stemness of alveolar type 2 cells. Science 359:1118–1123. 29420258 10.1126/science.aam6603 PMC5997265

[phy213831-bib-0040] Ortiz, L. A. , M. Dutreil , C. Fattman , A. C. Pandey , G. Torres , K. Go , et al. 2007 Interleukin 1 receptor antagonist mediates the antiinflammatory and antifibrotic effect of mesenchymal stem cells during lung injury. Proc. Natl Acad. Sci. USA 104:11002–11007. 17569781 10.1073/pnas.0704421104 PMC1891813

[phy213831-bib-0041] Pei, L. , M. Leblanc , G. Barish , A. Atkins , R. Nofsinger , J. Whyte , et al. 2011 Thyroid hormone receptor repression is linked to type I pneumocyte‐associated respiratory distress syndrome. Nat. Med. 17:1466–1472. 22001906 10.1038/nm.2450 PMC3210920

[phy213831-bib-0042] Planès, C. , M. Blot‐Chabaud , M. A. Matthay , S. Couette , T. Uchida , and C. Clerici . 2002 Hypoxia and beta 2‐agonists regulate cell surface expression of the epithelial sodium channel in native alveolar epithelial cells. J. Biol. Chem. 277:47318–47324. 12372821 10.1074/jbc.M209158200

[phy213831-bib-0043] Ritchie, M. E. , B. Phipson , D. Wu , Y. Hu , C. W. Law , W. Shi , et al. 2015 limma powers differential expression analyses for RNA‐sequencing and microarray studies. Nucleic Acids Res. 43:e47. 25605792 10.1093/nar/gkv007 PMC4402510

[phy213831-bib-0044] Rubenfeld, G. D. , E. Caldwell , E. Peabody , J. Weaver , D. P. Martin , M. Neff , et al. 2005 Incidence and outcomes of acute lung injury. N. Engl. J. Med. 353:1685–1693. 16236739 10.1056/NEJMoa050333

[phy213831-bib-0045] dos Santos, C. C. , S. Murthy , P. Hu , Y. Shan , J. J. Haitsma , S. H. J. Mei , et al. 2012 Network analysis of transcriptional responses induced by mesenchymal stem cell treatment of experimental sepsis. Am. J. Pathol. 181:1681–1692. 23083833 10.1016/j.ajpath.2012.08.009

[phy213831-bib-0046] Schmittgen, T. D. , and K. J. Livak . 2008 Analyzing real‐time PCR data by the comparative C(T) method. Nat. Protoc. 3:1101–1108. 18546601 10.1038/nprot.2008.73

[phy213831-bib-0047] Sidhu, S. S. , S. Yuan , A. L. Innes , S. Kerr , P. G. Woodruff , L. Hou , et al. 2010 Roles of epithelial cell‐derived periostin in TGF‐beta activation, collagen production, and collagen gel elasticity in asthma. Proc. Natl Acad. Sci. USA 107:14170–14175. 20660732 10.1073/pnas.1009426107 PMC2922596

[phy213831-bib-0048] Spaggiari, G. M. , A. Capobianco , S. Becchetti , M. C. Mingari , and L. Moretta . 2006 Mesenchymal stem cell‐natural killer cell interactions: evidence that activated NK cells are capable of killing MSCs, whereas MSCs can inhibit IL‐2‐induced NK‐cell proliferation. Blood 107:1484–1490. 16239427 10.1182/blood-2005-07-2775

[phy213831-bib-0049] Stegemann‐Koniszewski, S. , A. Jeron , M. Gereke , R. Geffers , A. Kröger , M. Gunzer , et al. 2016 Alveolar type II epithelial cells contribute to the anti‐influenza a virus response in the lung by integrating pathogen‐ and microenvironment‐derived signals. mBio 7:e00276. 27143386 10.1128/mBio.00276-16 PMC4959657

[phy213831-bib-0050] Stone, K. C. , R. R. Mercer , P. Gehr , B. Stockstill , and J. D. Crapo . 1992 Allometric relationships of cell numbers and size in the mammalian lung. Am. J. Respir. Cell Mol. Biol. 6:235–243. 1540387 10.1165/ajrcmb/6.2.235

[phy213831-bib-0051] Subramanian, A. , P. Tamayo , V. K. Mootha , S. Mukherjee , B. L. Ebert , M. A. Gillette , et al. 2005 Gene set enrichment analysis: a knowledge‐based approach for interpreting genome‐wide expression profiles. Proc. Natl Acad. Sci. USA 102:15545–15550. 16199517 10.1073/pnas.0506580102 PMC1239896

[phy213831-bib-0052] Thompson, B. T. , R. C. Chambers , and K. D. Liu . 2017 Acute respiratory distress syndrome. N. Engl. J. Med. 377:562–572. 28792873 10.1056/NEJMra1608077

[phy213831-bib-0053] Thorley, A. J. , P. A. Ford , M. A. Giembycz , P. Goldstraw , A. Young , and T. D. Tetley . 2007 Differential regulation of cytokine release and leukocyte migration by lipopolysaccharide‐stimulated primary human lung alveolar type II epithelial cells and macrophages. J. Immunol. 1950:463–473. 10.4049/jimmunol.178.1.463 17182585

[phy213831-bib-0054] Uehara, A. , Y. Fujimoto , K. Fukase , and H. Takada . 2007 Various human epithelial cells express functional Toll‐like receptors, NOD1 and NOD2 to produce anti‐microbial peptides, but not proinflammatory cytokines. Mol. Immunol. 44:3100–3111. 17403538 10.1016/j.molimm.2007.02.007

[phy213831-bib-0055] Ware, L. B. , and M. A. Matthay . 2002 Keratinocyte and hepatocyte growth factors in the lung: roles in lung development, inflammation, and repair. Am. J. Physiol. Lung Cell. Mol. Physiol. 282:L924–L940. 11943656 10.1152/ajplung.00439.2001

[phy213831-bib-0056] Whitsett, J. A. , L. M. Nogee , T. E. Weaver , and A. D. Horowitz . 1995 Human surfactant protein B: structure, function, regulation, and genetic disease. Physiol. Rev. 75:749–757. 7480161 10.1152/physrev.1995.75.4.749

[phy213831-bib-0057] Williams, A. E. , R. J. José , P. F. Mercer , D. Brealey , D. Parekh , D. R. Thickett , et al. 2017 Evidence for chemokine synergy during neutrophil migration in ARDS. Thorax 72:66–73. 27496101 10.1136/thoraxjnl-2016-208597 PMC5329051

[phy213831-bib-0058] Zamaraev, A. V. , G. S. Kopeina , E. A. Prokhorova , B. Zhivotovsky , and I. N. Lavrik . 2017 Post‐translational modification of caspases: the other side of apoptosis regulation. Trends Cell Biol. 27:322–339. 28188028 10.1016/j.tcb.2017.01.003

[phy213831-bib-0059] Zappia, E. , S. Casazza , E. Pedemonte , F. Benvenuto , I. Bonanni , E. Gerdoni , et al. 2005 Mesenchymal stem cells ameliorate experimental autoimmune encephalomyelitis inducing T‐cell anergy. Blood 106:1755–1761. 15905186 10.1182/blood-2005-04-1496

[phy213831-bib-0060] Zemans, R. L. , N. Briones , M. Campbell , J. McClendon , S. K. Young , T. Suzuki , et al. 2011 Neutrophil transmigration triggers repair of the lung epithelium via beta‐catenin signaling. Proc. Natl Acad. Sci. USA 108:15990–15995. 21880956 10.1073/pnas.1110144108 PMC3179042

