# Supplementary material for: Effects of bone marrow‐derived mesenchymal stromal cells on gene expression in human alveolar type II cells exposed to TNF‐α, IL‐1β, and IFN‐γ
Source: Physiol Rep. 2018 Aug 22;6(16):e13831. doi: 10.14814/phy2.13831 (PMC6105627; doi:10.14814/phy2.13831)
Supplement: Supplementary file 2 [file PHY2-6-e13831-s002.docx]

Supplementary table 1: Genes differentially expressed by ATII cells that were exposed to CytoMix alone vs. control. P-values were generated using the Bioconductor package limma, and they were adjusted for multiple testing using the Benjamini-Hochberg method.

Supplementary table 2: MSigDB Hallmark gene sets and canonical pathway gene sets up-regulated in ATII cells that were exposed to CytoMix alone vs. control. Enrichment p-values in each analysis were adjusted using the Benjamini-Hochberg method.

Supplementary table 3: MSigDB Hallmark gene sets and canonical pathway gene sets down-regulated in ATII cells that were exposed to CytoMix alone vs. control. Enrichment p-values in each analysis were adjusted using the Benjamini-Hochberg method.

Supplementary table 4: Genes differentially expressed by ATII cells that were exposed to MSCs plus CytoMix vs. CytoMix alone. P-values were generated using the Bioconductor package limma, and they were adjusted for multiple testing using the Benjamini-Hochberg method.

Supplementary table 5: MSigDB Hallmark gene sets and canonical pathway gene sets up-regulated in ATII cells that were exposed to MSCs plus CytoMix vs. CytoMix alone. Enrichment p-values in each analysis were adjusted using the Benjamini-Hochberg method.

Supplementary table 6: TNF-α signaling genes that are up-regulated with CytoMix or MSC exposure. This table lists genes that are up-regulated in ATII cells exposed to either CytoMix alone or CytoMix plus MSCs, and which are in the Hallmark gene set describing TNF-α signaling through NF-κB. We also show the overlap between the TNF-α genes that are up with CytoMix and those that are up with both CytoMix and MSCs.

Supplementary table 7: MSigDB Hallmark gene sets and canonical pathway gene sets down-regulated in ATII cells that were exposed to MSCs and CytoMix compared to CytoMix alone. Enrichment p-values were adjusted for multiple testing using the Benjamini-Hochberg method.
